# Numerical Model for Flexural Analysis of Precast Segmental Concrete Beam with Internal Unbonded CFRP Tendons

**DOI:** 10.3390/ma15124105

**Published:** 2022-06-09

**Authors:** Wutong Yan, Liangjiang Chen, Bing Han, Huibing Xie, Yue Sun

**Affiliations:** 1 China Railway Economic and Planning Research Institute Co., Ltd., Beijing 100038, China; chenliangjiang66@126.com; 2 School of Civil Engineering, Beijing Jiaotong University, Beijing 100044, China; bhan@bjtu.edu.cn (B.H.); hbxie@bjtu.edu.cn (H.X.); 3 China Building Technique Group Co., Ltd., Beijing 100013, China; 17121105@bjtu.edu.cn

**Keywords:** precast concrete segmental beam, internal unbonded tendons, CFRP, numerical model, flexural capacity, ductility

## Abstract

A new construction scheme was recently developed for precast segmental concrete beams by replacing steel tendons with internal unbonded carbon-fiber-reinforced polymer tendons. The discontinuous behaviors of the opening joints and unbonded phenomenon of tendons made their flexural behaviors more complicated than those of monolithic beams and members with bonded tendons. Currently, the knowledge on the structural performance of precast segmental concrete beams with internal unbonded carbon-fiber-reinforced polymer tendons is still limited. An efficient numerical model is urgently needed for the structural analysis and performance evaluation of this new construction scheme. In this paper, a new beam–cable hybrid model was proposed accounting for the mechanical behaviors of open joints and unbonded tendons. The numerical model was implemented in the OpenSees software with the proposed modeling method for joint elements and a newly developed element class for internal unbonded tendons. The effectiveness of the proposed model was verified by comparisons against two simply supported experimental tests. Then, the numerical model was employed to evaluate the flexural performance of a full-scale bridge with a span of 37.5 m. Compared with the precast segmental concrete beam with external steel tendons, the scheme with internal unbonded carbon-fiber-reinforced polymer tendons significantly improved the flexural capacity and ductility by almost 54.6% and 8.9%, respectively. The span-to-depth ratio and prestressing reinforcement ratio were the main factors affecting the flexural behaviors. With the span-to-depth ratio increasing by 23%, the flexural capacity decreased by approximately 38.6% and the tendon stress increment decreased by approximately 15.7%. With the prestressing reinforcement ratio increasing by 65.4%, the flexural capacity increased by 88.7% and the tendon stress increment decreased by approximately 25.2%.

## 1. Introduction

Precast segmental concrete beams (PSCBs) have become a popular construction option in recent decades due to their significant advantages in construction speed and quality control [1,2,3]. As the most critical component to ensure the integrity of the assembled concrete segments, the reliable prestressing scheme is of great concern for PSCBs.

Nowadays, steel tendons constitute the most widely adopted prestressing solution in PSCBs, including internal fully bonded steel tendons, external unbonded steel tendons, and hybrid tendons [4]. Numerous studies have been performed on the flexural and shear behaviors of steel tendon prestressed PSCBs [5,6,7,8,9,10]. The internal bonded steel tendons are beneficial to improving structural load-carrying capacity and ductility, but they are vulnerable to corrosion at the joint locations [11,12]. Once corrosion occurs, deterioration is inevitable, and the embedded steel tendons are almost impossible to be replaced. The application of external steel tendons is a solution option due to their property of being easier to handle, monitor, and replace during their service life [13]. However, the flexural capacity of PSCBs with external steel tendons is reduced due to the decreased tendon effective depth at the ultimate states [14,15,16,17]. A better prestressing scheme thoroughly considering both the tendon durability and structural load-carrying capacity is greatly needed for PSCBs.

Fiber-reinforced polymer (FRP) composites have the remarkable advantages of high strength and free corrosion [18,19], which are favorable for use in prestressing applications as alternatives to steel tendons. Previously, carbon-fiber-reinforced polymer (CFRP) tendons were usually applied to monolithic beams [20]. Le et al. first proposed the scheme of the precast segmental concrete beam with internal carbon-fiber-reinforced polymer tendons (PSCB-IUCFRPs) [21,22]. This new system has two main expected advantages compared with previous ones. First, the pipe grouting and epoxy joints are no longer needed, due to the noncorrosion property of the CFRP material, contributing to rapid construction and maintenance [23]. Second, the flexural capacity can be improved due to the better-maintained tendon eccentricity and high tensile strength of CFRP tendons. However, the structural performance of the PSCB-IUCFRP was only tested in two scaled experiments by Le et al. [21,22]. The knowledge on the flexural behaviors of the full-scale PSCB-IUCFRP is still limited. There is a need to develop the applicative numerical model and verify the structural performance of the full-scale PSCB-IUCFRP.

Compared with monolithic beams [20,24,25,26,27], the PSCB-IUCFRP system showed more complicated flexural behaviors, including the discontinuous behaviors of open joints and the unbonded phenomenon of the internal unbonded CFRP tendons. Le et al. [28] and Tran et al. [29] conducted the only numerical model for the PSCB-IUCFRP. In their models, concrete segments and internal tendons were modeled using the 3D solid finite elements (FE) model. The joint connection and unbonded behavior of tendons were modeled using contact elements. The proposed 3D FE method was verified against the experiments but came at a high computational cost. It was inconvenient for application to the full-scale beam. Lou et al. [30,31] and Pang et al. [32] proposed a higher-efficiency numerical model, in which the concrete beam was modeled by layered beam elements, and FRP tendons were modeled by the equivalent loads. However, their models could only be used for monolithic concrete beams, and two inadequacies were revealed. First, the discontinuous behaviors of the open joints were not considered. Then, the unbonded CFRP tendon was only modeled by equivalent loads by neglecting the stiffness, which decreased the numerical convergence for highly nonlinear solutions. To date, there is still no available high-efficiency numerical model for the structural analysis of the full-scale PSCB-IUCFRP.

In this paper, a new high-efficiency nonlinear analysis model is proposed for the analysis of the PSCB-IUCFRP, accounting for the discontinuous behaviors of open joints and the unbonded phenomenon of internal CFRP tendons. The concrete segment is modeled by the fiber beam element. The segmental joints are modeled by the “plain concrete” fiber beam element using the smeared strain method. The unbonded CFRP tendons are modeled by the proposed multi-node slipping cable element with complete formulations of the stiffness matrix and resistance vector, which improves the numerical convergency. The numerical model is implemented in the OpenSees software (version 2.5.0) [33] with newly developed element types and verified against experimental tests. Then, the numerical model is applied to evaluate the flexural capacity, ductility, and failure mode of a 37.5 m full-scale PSCB-IUCFRP. The effects of the main factors, including loading types, span-to-depth ratio, and prestressing reinforcement ratio, on the flexural behaviors of the PSCB-IUCFRP are discussed. To the best of the author’s knowledge, this is the first time to evaluate the flexural performance of the PSCB-IUCFRP in full-scale bridges.

## 2. Numerical Model Description

### 2.1. Modeling Schemes

**Figure 1:**
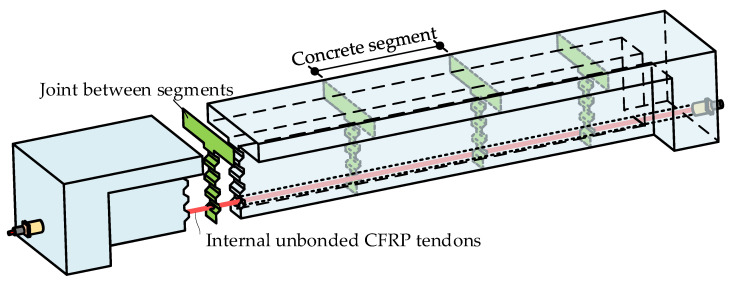
Schematic diagram of a PSCB prestressed with internal unbonded CFRP tendons.

The PSCB-IUCFRP system mainly comprises three components, namely, concrete segments, segmental joints, and internal unbonded CFRP tendons, as shown in Figure 1. The discontinuity of the opening joints and the unbonded phenomenon of prestressing tendons lead to more complicated flexural behaviors than monolithic or bonded prestressed concrete beams.

For the segmental joints, once decompressing at the dry joints or reaching the cracking strain at epoxy joints, the joints will open and the joint width will increase quickly due to the lack of bonded reinforcements. The segments adjacent to the open joints become discontinuous, which is the first issue that should be considered in the numerical model.

For the internal unbonded tendons, the friction forces between the unbonded tendons and the adjacent concrete are smaller than tendon traction [16,34]. Therefore, the frictionless assumption is usually adopted for unbonded tendons to simplify analysis [28,29,30]. With this assumption, internal unbonded tendons behave as a slipping cable with equal traction anywhere between two anchorages. The elongation of the unbonded tendons is member-dependent rather than exhibiting only section-dependent behavior. The interaction between internal unbonded tendons and concrete beams exists within the entire beam length. They have the same vertical deformation but different longitudinal deformation, which is the second issue to consider.

Tran et al. conducted the first numerical model study for the PSCB-IUCFRP using the 3D solid element FE model [29]. The CFRP tendon and concrete beam were modeled by solid elements and specified no-friction contact elements between them to model the unbonded phenomenon. The joints were simulated using the contact elements with a friction coefficient of 0.7 to model the discontinuous behaviors of the opening joints. The solid FE model showed good accuracy but came at a high computational cost due to the large amounts of elements and complex contact interactions that existed, decreasing the computational efficiency and convergence for the analysis of full-scale beams.

In this paper, we propose a one-dimensional beam–cable hybrid element model to improve the analysis efficiency and convergence. Based on the experimental tests and observations [21,22], the following reasonable assumptions are considered:

- The structural deformation and capacity are dominated by flexural behaviors. The effects of the shear deformation on the beam are neglected.
- The direct shear failure of joints will not occur before the structural flexural failure.
- Previous tests [16,34] showed that the friction between the unbonded tendons and surrounding concrete is usually small. For simplification, we neglect the friction effects in this work and assume that the traction of the internal unbonded tendon is constant between the anchorages [28,29,30].

**Figure 2:**
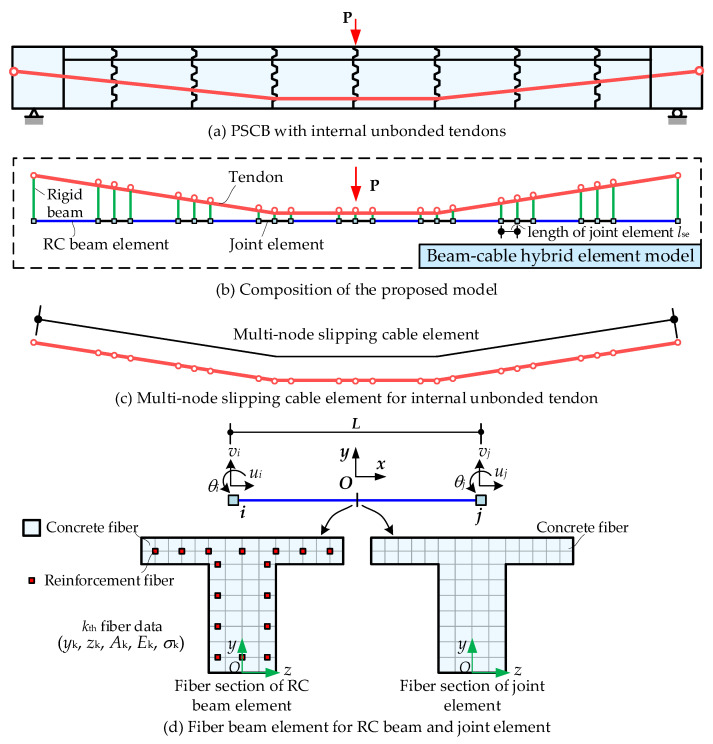
Modeling schemes of the proposed model.

With the above assumptions, the proposed modeling scheme is established, as shown in Figure 2. The main characteristics of the proposed model are concluded as follows:

(1) The segmental concrete beam is modeled by the nonlinear fiber beam element to improve the analysis efficiency. In particular, the segmental joints are modeled by the “plain concrete” fiber beam element with a specified element length *l_se_*. The discontinuous joint behaviors are converted into the deformation of equivalent continuum elements based on the smeared strain method, which is described in Section 2.2.
(2) The newly developed multi-node slipping cable element is employed to model the unbonded behavior of internal unbonded tendons. The deformation compatibility is satisfied and the strain is equal anywhere within the element, as presented in Section 2.3.
(3) The interaction between the concrete beam and unbonded tendon is simulated by the rigid beam, which transfers the translational and rotational deformations of beam nodes to the translation of tendon nodes, as presented in Section 2.4.

The proposed modeling method and derived FE formulations are implemented in the OpenSees software [33] for simulation. The mechanical characteristics of the PSCB-IUCFRP, including nonlinear material behaviors, discontinuous joint behaviors, and the unbonded phenomenon of CFRP tendons, are all thoroughly considered using one-dimensional element types, improving the analysis efficiency.

### 2.2. Fiber Beam Element for Segmental Concrete Beam

**Figure 3:**
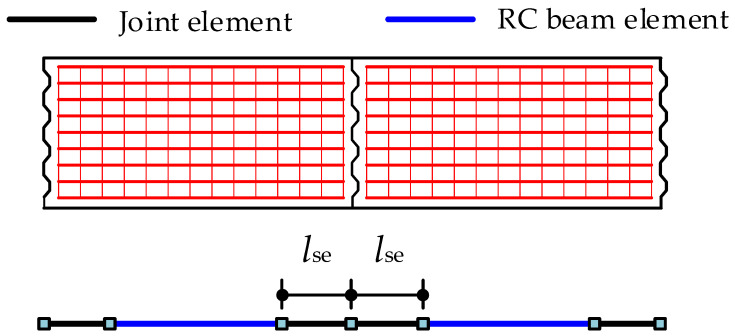
Modeling method of concrete beam segments.

The modeling method for the segmental concrete beam in this paper is shown in Figure 3, in which each concrete beam segment can be generally modeled by three elements. Two joint elements are arranged at two ends of the segment and one RC beam element is placed in the middle. Both the joint element and the RC beam element are modeled by the fiber beam element in the OpenSees software [35]. The cross-section of this element is divided into many small regions, which are called “fibers” in the OpenSees software [35]. The region for concrete is called “concrete fiber” and the region for reinforcement is called “reinforcement fiber”, as shown in Figure 2d. Each fiber contains a uniaxial material constitutive model, an area, and centroid coordinates (*y*, *z*). For the Euler–Bernoulli beam, the axial deformations of the fibers within the cross-section satisfy the plane section assumption.

The nonlinear state updating process of the element can be concluded as follows. With the specified element deformation, the axial deformation of each fiber in the cross-section is first calculated. Then, the stress and tangent modulus of each fiber are updated according to the uniaxial nonlinear constitutive model. Then, the sectional resistance and stiffness can be calculated by the sum of all of the fibers’ axial forces and tangent stiffness. The element resistance and stiffness are subsequently calculated with the sectional resistance and stiffness using the Gauss–Lobatto numerical integration method. The detailed FE formulations and algorithmic implementation of this element can be seen in [36]. The corotational coordinate transformation is adopted to consider the geometrically nonlinear behavior of the element.

**Figure 4:**
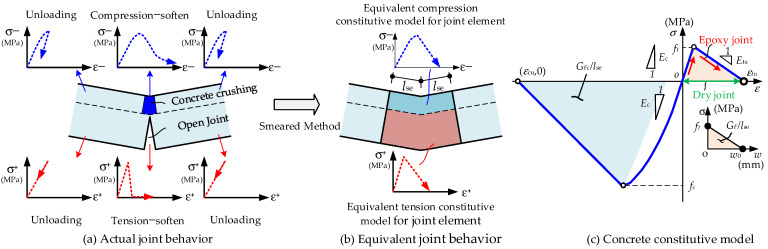
Smeared strain method and concrete constitutive model for joint element.

The main issue that should be emphasized here is the difference in fiber section definition between the joint and RC beam elements, as shown in Figure 2d. The fiber section of the RC beam element includes concrete fibers and reinforcement fibers, while in joint elements, only concrete fibers are included as reinforcements are cut-off at the joint locations. With the increasing load up to ultimate states, the bottom fiber of the joint will be open, and the compression region will be crushed. In the numerical model, we define the length of the joint element as *l_se_*. The actual deformations of the joint and the adjacent concrete (as shown in Figure 4a) are averagely smeared into the joint element. The concrete constitutive model in the joint element is equivalently modified according to the fracture and crushing band theory [37,38], as shown in Figure 4b. Based on this equivalent smeared strain method, the effects of the joint element length can be normalized. Generally, specifying *l_se_* as no greater than one-fourth of the segment length is applicable.

According to the concept of smeared strain, the unified concrete constitutive model is adopted, as shown in Figure 4c. For epoxy joints, the tensile strength of the fiber in the joint element is set to be equal to that of the concrete segments. For dry joints, the tensile strength of the fiber is set to be zero. The shadows under the tensile and compressive curves in Figure 4c denote fracture energy *G_F_* (N/mm) and crushing energy *G_Fc_* (N/mm) of the concrete per unit length, respectively. The slopes of the compression-softening and tension-stiffening curves are adjusted according to *G_Fc_* (N/mm) and *G_F_* (N/mm), as shown in Equations (1) and (2). The values of *G_Fc_* (N/mm), *G_F_* (N/mm), and *G_F_*_0_ (N/mm) are calculated according to the specifications of CEB-FIP [39]. Then, the crack and crushing bands are smeared into the whole element length *l_se_* (mm) to reach normalization. Furthermore, the joint opening width can be approximatively predicted by the product of tensile strain and joint element length *l_se_* (mm). In this way, the discontinuous joint behaviors are converted to the equivalent “continuous behavior”.

(1) $$\varepsilon_{cu}=2G_{Fc}/\left(f_{c}l_{se}\right)+\varepsilon_{c0},\;G_{Fc}=8.8\sqrt{f_{c}}$$

(2) $$\varepsilon_{tu}=2G_{F}/\left(l_{se}f_{t}\right),\;G_{F}=G_{F0}{\left(f_{c}/10\right)}^{0.7}$$

### 2.3. Multi-Node Slipping Cable Element for Internal Unbonded Tendons

In the traditional FE model, the prestressing tendons are usually modeled by the truss element, which is a 2-node line element. For internal unbonded prestressed members, the tendon needs to be divided into many truss elements to apply the interaction with the beam along the whole span. However, the strain compatibility cannot be satisfied by the traditional truss elements, which are not suitable for the simulation of the free-slip behaviors of internal unbonded tendons.

**Figure 5:**
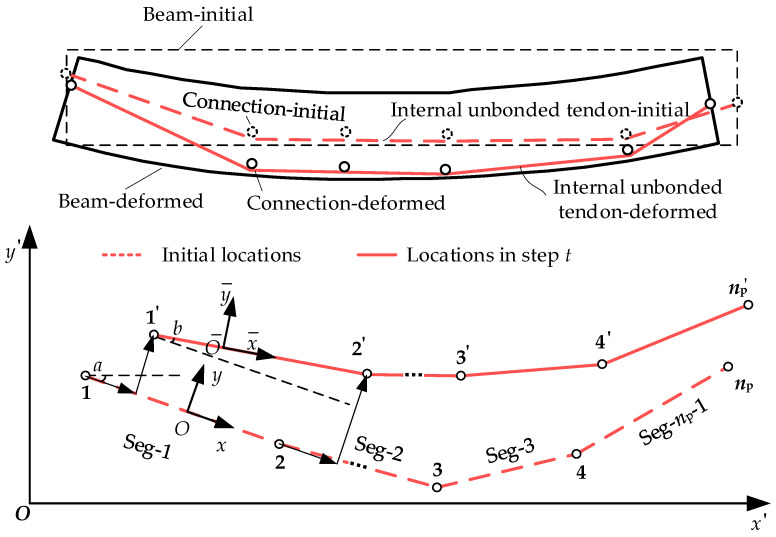
Schematic diagram of multi-node slipping cable element for internal unbonded tendon.

To model the free-slip effects of internal unbonded tendons, a new type of element called the multi-node slipping cable element is proposed by the authors. The schematic diagram of the proposed element is shown in Figure 5. The element consists of several nodes and segments, and the nodal as well as segmental numbers are arbitrary without limitation. In this paper, we assume the slipping cable element as a configuration with *n*_p_ nodes and *n*_p−1_ segments. Each node has two translational DOFs, and each segment can only transmit only axial force. The axial forces in the segments are equal anywhere.

At step *t*, we express the element displacement vector **U***_t_* as Equation (3).

(3) $$U_{t}={\left[\begin{array}{cccc} u_{1,t}^{} & u_{i,t}^{} & \cdots & u_{n_{p},t}^{} \end{array}\right]}^{T}{}_{2n_{p}\times 1},$$

where $u_{i,t}^{}=\left[\begin{array}{cc} u_{i,t} & v_{i,t} \end{array}\right]\left(i=1∼n_{p}\right)$ is the displacement vector of node *i* at step *t*; $u_{i,t}$ and $v_{i,t}$ denote the displacement along the *x* and *y* direction of the global coordinate system, respectively.

For each segment, we define the element local coordinate system $\bar{x}\bar{O}\bar{y}$ along the deformed element axis based on the corotational theory. Then, the element displacement vector $U_{t}^{l}$ in the local coordinate system can be denoted as Equation (4).

(4) $$\begin{array}{l} U_{t}^{l}=RP_{t}U_{t}\\ P_{t}^{}={\left[\begin{array}{ccccccc} -1 & 0 & 1 & & & & \\ & -1 & 0 & 1 & & & \\ & & -1 & 0 & 1 & & \\ & & & \ddots & \ddots & \ddots & \\ & & & & -1 & 0 & 1 \end{array}\right]}_{\left(2n_{p}-2\right)\times \left(2n_{p}\right)},\;R=diag{\left(\left[\begin{array}{cccc} r_{1} & r_{j} & \cdots & r_{n_{p}-1} \end{array}\right]\right)}_{\left(2n_{p}-2\right)\times \left(2n_{p}-2\right)}\\ r_{j}=\left[\begin{array}{cc} \cos X & \cos Y\\ -\cos Y & \cos X \end{array}\right],\;\cos X=\frac{x_{j+1,0}-x_{j,0}}{l_{j,0}},\;\cos Y=\frac{y_{j+1,0}-y_{j,0}}{l_{j,0}} \end{array}$$

Then, the tendon strain at step *t* can be calculated using the definition of the engineering strain, as shown in Equation (5).

(5) $$\begin{array}{l} \varepsilon^{t}=L_{t}/L_{0}=U_{t}^{T}P_{t}^{T}{ϒ}_{t}\left[P_{t}U_{t}+R^{T}L_{0}\right]/L_{0}\\ {ϒ}_{t}=diag\left(\left[\begin{array}{cccc} l_{1,t}^{-1}I_{2\times 2} & l_{j,t}^{-1}I_{2\times 2} & \cdots & l_{{}_{n_{p}-1},t}^{-1}I_{2\times 2} \end{array}\right]\right)\\ L_{0}={\left[\begin{array}{cccc} L_{1,0}^{T} & L_{j,0}^{T} & \cdots & L_{n_{p}-1,0}^{T} \end{array}\right]}^{T},\;L_{j,0}={\left[\begin{array}{cc} l_{j,0} & 0 \end{array}\right]}^{T} \end{array}$$

where *L*_0_ denotes the initial sum length of all segments and *L_t_* denotes the sum length of all segments at step *t*.

Through the virtual work equations and variational derivation, the stiffness matrix **K***_t_* and resistance vector **f***_t_* of the slipping cable element can be obtained and expressed as Equations (6) and (7), respectively. $k_{t}^{g}$ and $k_{t}^{m}$ denote the geometric and material stiffness matrix of the element, respectively. *E*_p_ is the tangent modulus of the material and *A*_p_ is the cross-sectional area of the tendon. $\sigma^{t}$ denotes the stress in current analysis step *t*.

(6) $$\begin{array}{l} K_{t}=k_{t}^{g}+k_{t}^{m}\\ k_{t}^{g}=\sigma^{t}A_{p}P_{t}^{T}\left({ϒ}_{t}-\Gamma_{t}\right)P_{t}\\ k_{t}^{m}=E_{p}A_{p}/L_{0}P_{t}^{T}\Gamma_{t}R^{T}L_{0}L_{0}^{T}R{ϒ}_{t}P_{t} \end{array}$$

(7) $$f_{t}=\sigma^{t}A_{p}L_{0}^{T}R\Gamma_{t}P_{t}^{}f_{t}=\sigma^{t}A_{p}L_{0}^{T}R\Gamma_{t}P_{t}^{}$$

In the current OpenSees version, there are no suitable element types for the simulation of internal unbonded tendons. Based on the above-derived equation, the slipping cable element is added into the OpenSees software by the authors as the newly developed element type. Meanwhile, the *Tcl* command interpreters are also programmed into OpenSees for the definition of the element. It should be pointed out that the element and developed procedure have no limitation on the tendon shape profiles.

### 2.4. Rigid Beam Connection

**Figure 6:**
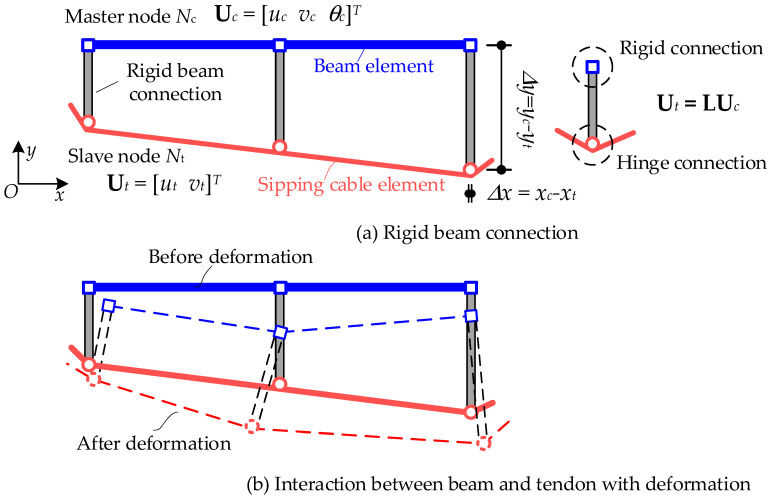
Rigid beam connection between beam and tendon.

The interactions between the concrete beam and tendons are modeled by a rigid beam connection, as shown in Figure 6. The node of the concrete beam has three degrees of freedom (DOFs), and the nodal displacement vector is denoted as **u***_c_* = [*u_c_*, *v_c_*, *θ_c_*]*^T^*. The node of the tendon element has two DOFs, whose displacement vector is denoted as **u***_t_* = [*u_t_*, *v_t_*]*^T^*. Taking the concrete beam node as the master node and the tendon node as the slave node, the rigid beam connection builds a transformation of displacements of the beam node to the displacements of the tendon node. The transformation relation of the rigid beam is presented in Equation (8). ∆*x* and ∆*y* denote the projection distances from the master node to the slave node in the *x* and *y*-directions, respectively.

(8) $$u_{t}=\left[\begin{array}{ccc} 1 & 0 & \Delta y\\ 0 & 1 & -\Delta x \end{array}\right]u_{c}$$

### 2.5. Constitutive Model

The nonlinear constitutive models of materials used in this paper are presented as follows:

(1) Concrete Model

The concrete model for the RC beam element and joint element uses a parabolic-ascending linear-descending form for the compressive stress–strain relation. The tensile stress–strain relation is modeled by a linear-elastic before cracking and linear-descending form after cracking. The diagram can be seen in Figure 4c. *f_t_* (MPa) and *f_c_* (MPa) are the tensile and compression strength of concrete, respectively. ε*_tu_* and ε*_cu_* denote the ultimate tensile and compression strain of concrete, respectively. *E_c_* (MPa) represents the initial modulus of concrete. For dry joint elements, the tensile strength of concrete is set to be zero and the built-in material Concrete01 in OpenSees without tension is adopted. For the epoxy joint, the built-in material Concrete02 in OpenSees is used and the tension softening stiffness is adjusted by the fracture energy, as presented in Equation (2). The compression-soften slope is adjusted by the crushing energy, as presented in Equation (1).

(2) Reinforcements Model

**Figure 7:**
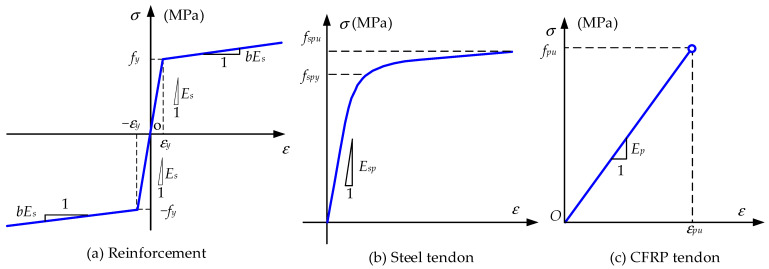
Constitutive model of reinforcement and tendons.

The Steel01 Model in the OpenSees software is adopted to model the material behaviors of reinforcements, as shown in Figure 7a. The model is linear-elastic before reaching the yield strain and then linear strain-hardening up to ultimate stress. *E_s_* (MPa) denotes the initial elastic modulus and *f_y_* (MPa) is the yield strength of reinforcement. *b* represents the strain-hardening ratio and is set to be 0.005 in this paper. The reinforcements model is only used in the RC beam element.

(3) Steel tendon

The material behavior of the prestressing steel tendon is modeled by the Steel02 model in OpenSees, as shown in Figure 7b. *f_spy_* (MPa) is the yield strength of the steel tendon. *E_sp_* (MPa) denotes the initial elastic modulus and *b_sp_* represents the strain-hardening ratio. In this paper, *b_sp_* = 0 and *R_0_* = 18 are employed in the Steel02 model definition and the default values are used for the other parameters (see http://opensees.berkeley.edu/ (accessed on 1 March 2020)). At the same time, the initial prestress *σ_pe_* (MPa) is defined in the Steel02 model.

(4) CFRP tendon

The CFRP tendon has no yield states and behaves linear-elastically until rupture. The adopted material model for the CFRP is shown in Figure 7c. In OpenSees, there is no available material model for the CFRP. Therefore, we develop the new material constitutive model for the CFRP, which is linear elastic for a strain less than *ε_pu_* and stress, and zero otherwise.

### 2.6. Novelty of the Proposed Model

Compared with previous studies, the novelty of the proposed model can be divided into three aspects as follows.

- This work proposes the first one-dimensional numerical model for the newly developed precast segmental concrete beam with internal unbonded CFRP tendons. Compared with the solid element model, the analysis efficiency and numerical convergence can be improved.
- Accounting for the discontinuous behaviors of the opening joint, a novel modeling idea, the “plain concrete” fiber beam element with the smeared strain method, is proposed.
- Accounting for the unbonded phenomenon of the internal unbonded tendon, a multi-node slipping cable element is proposed. In contrast to the equivalent load method, the complete formulations containing resistance and stiffness matrices are strictly derived, improving the numerical convergence for highly nonlinear solutions. The derived formulations are further developed in the OpenSees software as a newly developed element class.

## 3. Verification of Numerical Model

### 3.1. Verification for Tests of Monolithic Beam Prestressed with Internal Unbonded CFRP Tendon

**Figure 8:**
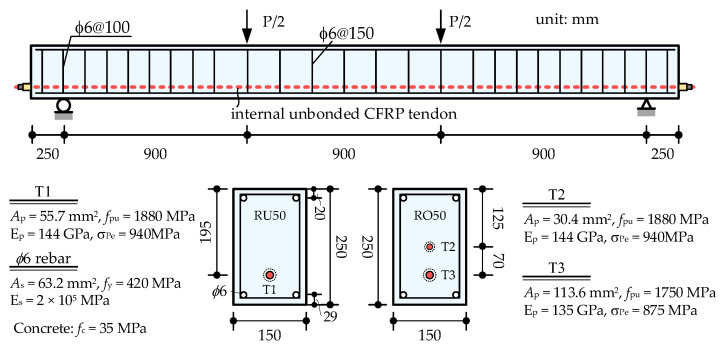
Design details of specimens RU50 and RO50 (unit: mm).

Heo et al. tested the flexural behaviors of the monolithic beam prestressed with the internal unbonded CFRP tendon [24,40], in which the specimens RU50 and RO50 were designed with the same dimensions but different prestressing reinforcement ratios. These two specimens are employed here to verify the effectiveness of the proposed model for the simulation of internal unbonded tendons. Figure 8 shows the structural details and material parameters of RU50 and RO50. In the numerical model, the concrete beam is modeled by 18 fiber beam elements of 150 mm in length, and the internal tendon is modeled by only one slipping cable element. Prestressing and self-weight are applied to the beam first, and then the external load is applied up to failure.

**Figure 9:**
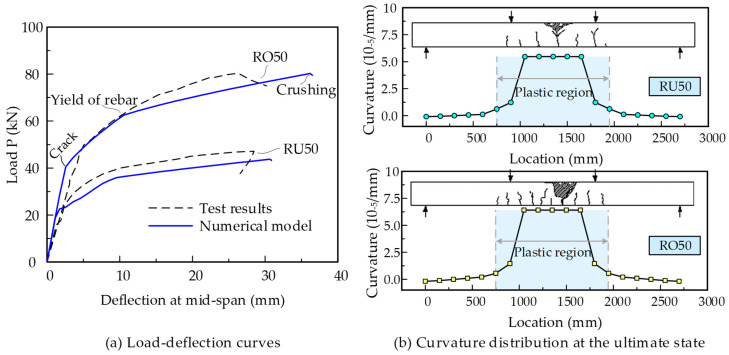
Analysis results for specimens RU50 and RO50.

The analysis results and the comparisons with the tests are shown in Figure 9. Figure 9a shows the load versus mid-span deflection curves. The specimen BO50, with larger prestressing reinforcement ratios, shows greater cracking load and flexural capacity than BU50. The predicted deviations in the flexural capacity for BU50 and BO50 are −7.3% and −0.15%, respectively. The numerical model shows fairly good agreements with the tests overall. Meanwhile, it should be noted that the load–deflection curves of the monolithic beam with internal unbonded CFRP tendons show three feature points, namely, cracking, the yield of rebar, and concrete crushing. The sectional curvature at the ultimate state is extracted from the numerical results and the distributions along the beam length are shown in Figure 9b. It can be seen that a plastic region with a certain length is formed at the mid-span of both RU50 and RO50. The numerical model shows similar failure modes to the tests.

### 3.2. Verification for Tests of PSCBIU-CFRP

**Figure 10:**
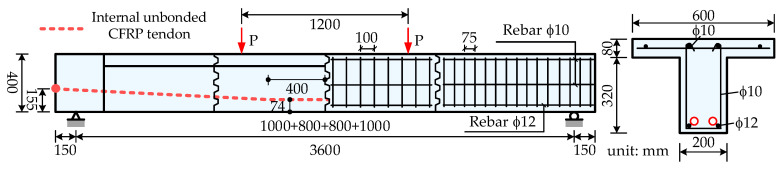
Structural dimensions of specimens C2 and C4 (unit: mm).

Le et al. [21,22] conducted the first experiments on the flexural behavior of a precast segmental concrete beam with internal unbonded CFRP tendons. The specimens named C2 and C4 are analyzed to verify the numerical model. The structural dimensions are shown in Figure 10 and the material parameters of the rebar and tendon are listed in Table 1. Two ϕ12 rebars are employed for bottom longitudinal reinforcement. Four ϕ10 rebars are placed at the top slab. ϕ10 reinforcements are used as the steel stirrups, with a 100 mm spacing for the middle segments and 75 mm for the end segments. The cylinder compressive strength of the concrete is 44.0 MPa on the testing day. The specimens C2 and C4 adopt the multiple shear-keyed epoxied joints, and the pre-tension control stress is set as 0.4 *f_pu_* for the CFRP tendon. After the completion of post-tensioning, specimen C2 is grouted as the bonded members and specimen C4 is not grouted as the unbonded PSCBs.

**Figure 11:**
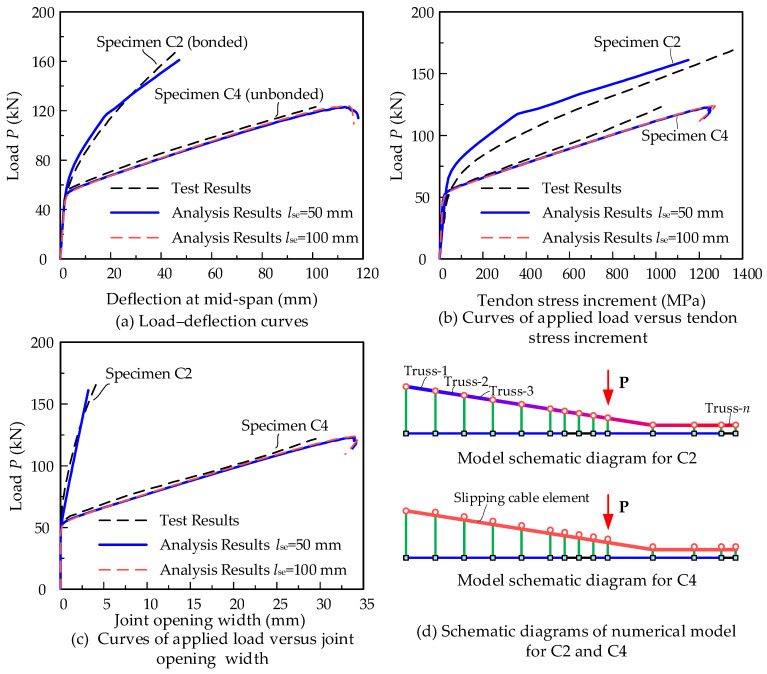
Analysis results for specimens C2 and C4.

Based on the structural dimensions, two different models are built using OpenSees software. In the numerical model, the concrete segment element is modeled by the joint and RC beam element presented in Section 2.2. The length of the joint element is set as 100 mm and the concrete constitutive model is modified by the mentioned smeared strain method. The main differences between the two models are the modeling method for the CFRP tendons. For specimen C2, the bonded CFRP tendon is modeled by several traditional truss elements, and the tendon nodes are connected to the beam element by the rigid beam. Under loading states, the forces of the tendon elements in C2 are different and the maximum tendon forces usually occur in the tendon element adjacent to the failure region. For specimen C4, the unbonded CFRP tendon is modeled by the proposed slipping cable element. The tendon force is equal anywhere in the whole tendon. The analysis results and comparisons with the tests are shown in Figure 11. Figure 11a shows the curves of the applied load versus the mid-span deflection. Figure 11b shows the curves of the applied load versus the tendon stress increment. The tendon stress increment is equal within the internal unbonded tendon in specimen C4. For specimen C2, the tendon stress increment is obtained from the middle segment where the tendon stress is the maximum. Figure 11c shows the curves of the applied load versus the joint opening width at mid-span. The schematic diagrams of the numerical models for C2 and C4 are shown in Figure 11d.

Overall, the analysis results show good agreements with the tests of structural capacity, deformation, tendon stress increments, and joint opening width. The bonded prestressed member C2 has a larger capacity but the unbonded prestressed member C4 shows better ductility. The conventional truss element could only be used for the simulation of the bonded CFRP tendon and the proposed slipping cable element could be employed to analyze the effects of the unbonded phenomenon. Moreover, the joint element length is taken as a discussed parameter. With the normalized constitutive model based on the fracture and crushing energy, the analysis results of models with joint element lengths of *l_se_* = 50 mm and *l_se_* = 100 mm are almost the same. It can be concluded that the proposed method can be effectively used to model the joint behavior and the effects of the joint element length can be eliminated.

**Figure 12:**
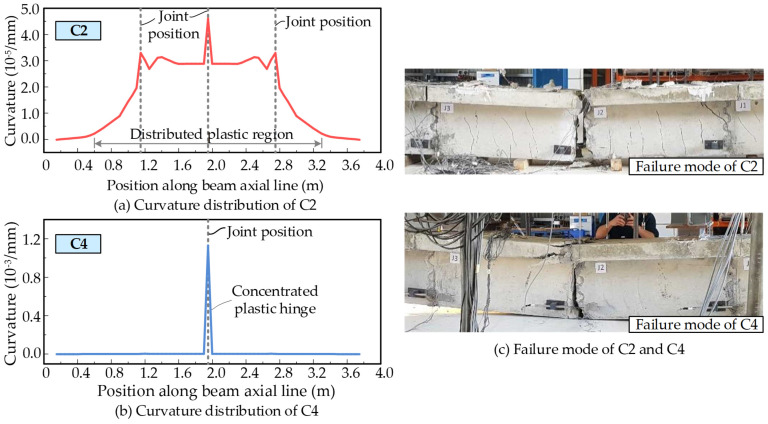
Failure mode comparisons for specimens C2 and C4.

To compare the failure mode differences, the curvature distributions along the beam axial line are extracted from the last analysis step and plotted in Figure 12. The comparisons show that the bonded member C2 has a certain length of the plastic region at mid-span, as with the monolithic beam in Figure 8. However, this is quite different for the unbonded beam C4. Figure 12b shows that a concentrated plastic hinge is formed at the mid-span of C4 and the other regions are almost elastic. The PSCBIU-CFRP shows different behaviors to the bonded beam.

However, the specimens by Le et al. [21,22] have only one joint at mid-span, which is different from the practice bridge. The flexural performances should be further evaluated in the full-scale bridge and the proposed model could be employed as a highly efficient analysis tool.

## 4. Flexural Performance Evaluation of PSCBIU-CFRP in Full-Scale Bridge

### 4.1. Flexural Performance Evaluation

Compared with the scaled beam, the full-scale PSCB consists of more segments with larger dimensions. The scale of the shear keys is very small compared with the concrete segment length, requiring significant numbers of elements in the 3D solid model during the mesh generation. The application of the 3D solid element model to the full-scale bridge is sometimes limited. The verified and high-efficiency numerical model in this paper provides an optional analysis tool for evaluating the flexural performance of the full-scale PSCBIU-CFRP system.

**Figure 13:**
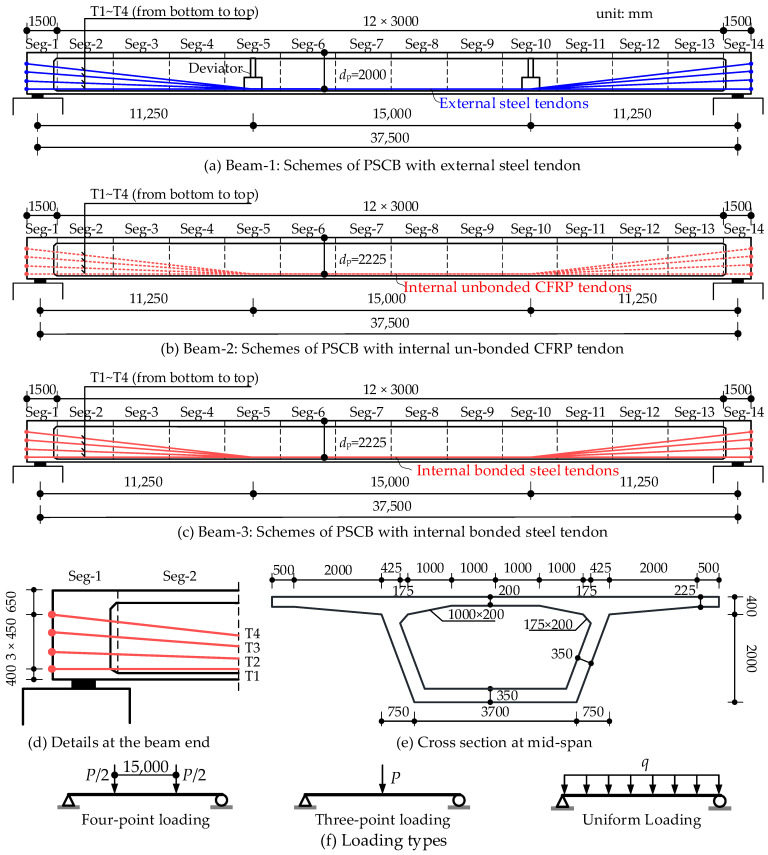
Structural dimensions of the numerical beam (unit: mm).

In this section, three different prestressing schemes for PSCBs are discussed, namely, the widely used external unbonded tendons, internal fully bonded tendons, and the newly developed internal unbonded CFRP tendons. The models are loaded by four-point loading (as shown in Figure 13f) up to failure; then, the flexural capacity and ductility of the different schemes are compared.

Figure 13a,b present the structural design details of PSCBs with external steel tendons (called Beam-1) and internal unbonded CFRP tendons (called Beam-2), respectively. Meanwhile, Beam-3 is designed to have the same structural dimensions as Beam-2, but replaces the internal unbonded CFRP tendons with internal bonded steel tendons (as shown in Figure 13c). The supported span is 37.5 m, consisting of 14 concrete box beam segments and prestressed by 8 tendons with symmetrical layouts. The dimensions of the cross-section are shown in Figure 13e. There are 120 and 60 conventional rebars ϕ12 in the top and bottom flanges, respectively. Furthermore, ϕ10 reinforcements are placed in the segment at 150 mm spacing to increase the shear strength of the segments. The concrete strength is 40.0 MPa, and the other material properties are listed in Table 2.

**Figure 14:**
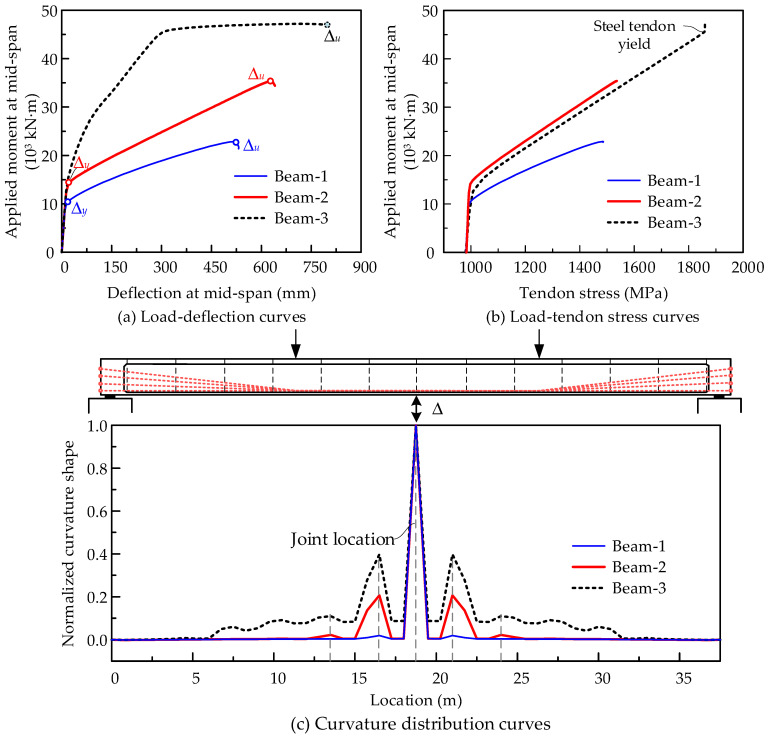
Performance comparisons of PSCB with different prestressing schemes.

The calculated flexural capacity, deformation, and tendon stress of the three models are compared in Figure 14, and the results are listed in Table 3. Some observations are concluded below:

(1) As shown in Figure 14a, the load–deflection curves of these beams show different shape styles. For Beam-3, prestressed with internal fully bonded steel tendons, the load–deflection curve is a three-broken-line shape with feature points of joint opening, steel tendon yield, and concrete crushing. However, for Beam-2 and Beam-1, the load–deflection curve shape is a double broken line with feature points of joint opening and concrete crushing.
(2) The beam with external steel tendons (Beam-1) shows a minimum flexural capacity of 22.9 × 10^3^ kN·m. The beam with internal fully bonded steel tendons (Beam-3) shows a maximum flexural capacity of 47.2 × 10^3^ kN·m. The application of the CFRP in the PSCB as internal unbonded prestressing significantly improves the flexural capacity. The ultimate capacity of 35.4 × 10^3^ kN·m increases by 54.6% compared with Beam-1 and is close to that of Beam-3, with only a 25% decrease. The comparisons show that the PSCB-IUCFRP system can be taken as the substitute for an external prestressed PSCB to improve structural capacity.
(3) Following the adopted ductility indices defined in the references [21,22], *μ* = *∆_u_*/*∆_y_*, the structural ductility is evaluated and compared. *∆_y_* is the deflection at mid-span when the joints are open. *∆_u_* is the deflection at mid-span when reaching the maximum load. The calculated ductility indices are listed in Table 3. All of the beams show good structural ductility, and the PSCB-IUCFRP system increases ductility indices by 8.9% compared with the external prestressed beam (Beam-1).
(4) The tendon stress along with the increasing applied load is shown in Figure 14b, in which the results of Beam-3 are from the tendon at mid-span. The tendon stress increments are all small before joint opening and then increase quickly once reaching the cracking load. At the ultimate state, the steel tendon in Beam-3 has been yielded and tendons in the unbonded beam Beam-1 and Beam-2 are still inelastic. The tendon stress increments of Beam-2 are larger than those of Beam-1. Meanwhile, the effective depth of the tendon in Beam-1 reduces by 201.7 mm due to the second-order effects, leading to a decrease in flexural capacity compared with Beam-2.
(5) Figure 14c shows the curvature distribution curves along the beam length in the ultimate state. In Beam-1, the curvature is mainly concentrated at mid-span joints and the other regions are almost still elastic. However, for Beam-2 and Beam-3, the deformations spread to the other joints, increasing the tendon stress and further flexural capacity.

### 4.2. Parametric Study

Taking Beam-2 in Figure 13b as the reference beam, the effects of loading types, span-to-depth ratio *L*/*d*_p_, and prestressing reinforcement ratio *ρ*_p_ = *A*_p_/*A*_c_ on the flexural performance are discussed.

(1) Loading types

**Figure 15:**
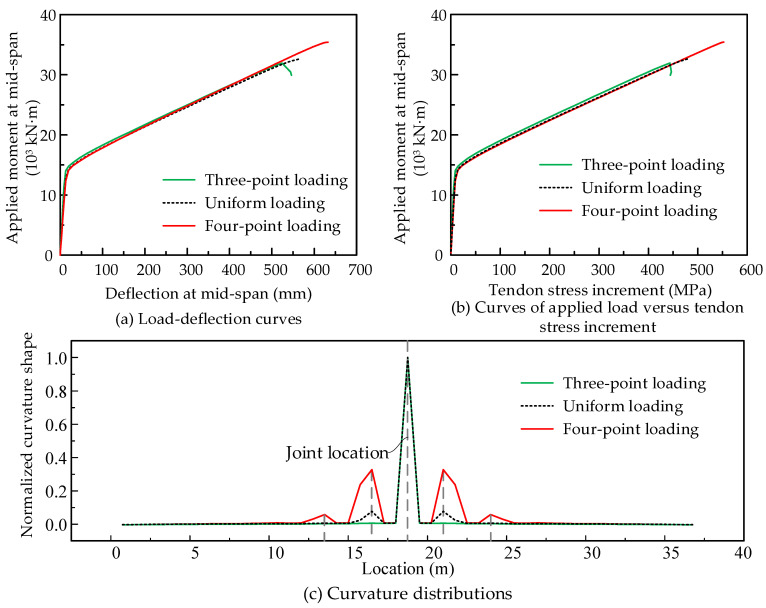
Effects of loading types on the flexural behaviors of PSCBIU-CFRP.

Three loading types as shown in Figure 13e are considered in the simulation and the analysis results are shown in Figure 15. We find that the loading types have certain effects on the flexural capacity. The model under three-point loading obtains the minimum flexural capacity. The maximum capacity can be carried under four-point loading states. This finding differs from the conclusions of Le et al. [21,22]. In their analysis model, there was only one joint placed at mid-span, and no additional joint opened under any loading types. However, more joints exist in the practice bridge, and the joint opening may not only occur at one joint. In this simulation, under four-point loading, more joints open in the pure bending region, as shown in Figure 15c. The joint opening width significantly influences the tendon stress increment (Figure 15b) and further increases the capacity.

(2) Span-to-depth ratio *L*/*d*_p_

**Figure 16:**
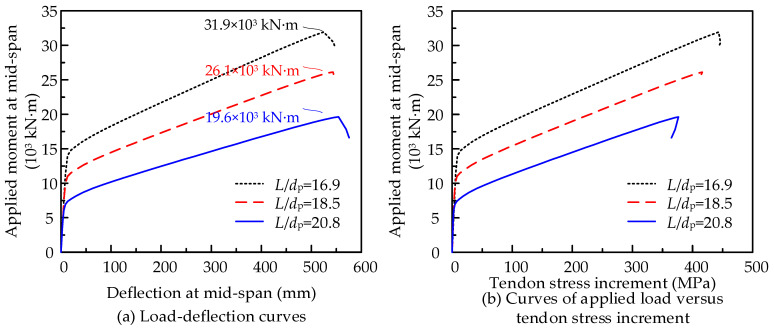
Effects of span-to-depth ratio *L*/*d*_p_ on the flexural behaviors of PSCBIU-CFRP.

Based on the design details of Beam-2 in Figure 13b, the tendon effective depth *d*_p_ varies from 2.025 and 2.225 to 1.8 m; then, beams with a span-to-depth ratio *L*/*d*_p_ of 16.85, 18.5, and 20.8 are obtained to discuss the effects on the flexural performances. All of the models are applied with three-point loading up to failure and the results are shown in Figure 16. With *L*/*d*_p_ increasing by 23%, the flexural capacity decreases by about 38.6% and the tendon stress increment decreases by about 15.7%. This reveals that a smaller span-to-depth ratio *L*/*d*_p_ is conducive to flexural performance.

(3) Prestressing reinforcement ratio *ρ*_p_

**Figure 17:**
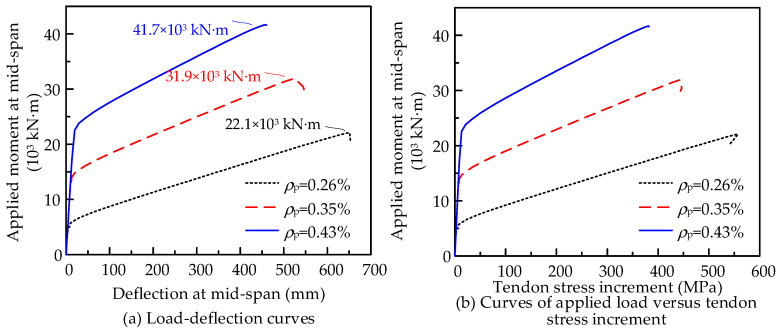
Effects of *ρ*_p_ on the flexural behaviors of PSCBIU-CFRP.

Based on the referenced beam in Figure 13b, the area of the tendon is taken as the variable to discuss the effects of *ρ*_p_ on the flexural performance. The cross-sectional area per tendon is set to 1575, 2100, and 2625 mm^2^, respectively. Three models are built with prestressing reinforcement ratios *ρ*_p_ of 0.26%, 0.35%, and 0.43%. Under three-point loading states, the flexural behaviors are analyzed and the results are presented in Figure 17. With *ρ*_p_ increasing by 65.4%, the flexural capacity increases by 88.7%. However, we also observe that the ultimate stress increment and the structural ductility decrease by 25.2% and 29.6%, respectively.

## 5. Conclusions

In this study, a new numerical model was proposed for the flexural analysis of a precast segmental concrete beam prestressed with internal unbonded CFRP tendons (PSCB-IUCFRPs). The modeling scheme, finite element formulations, and element implementation were presented. Two scaled experimental tests, namely, two monolithic beams and two segmental beams with internal unbonded CFRP tendons, were performed to verify the effectiveness of the proposed model. The verified numerical model was then applied to evaluate the flexural performance of a full-scale PSCB-IUCFRP with a span of 37.5 m. The influences of loading types, span-to-depth ratios, and prestressing reinforcement ratios on the flexural behaviors were further discussed. The following conclusions could be drawn:

1. The comparisons of two scaled experimental tests showed that the opening joint and the bonded condition of the tendons had significant influences on the flexural behaviors of the beam. The opening joint led to the concentrated plastic deformation at the joint location. The unbonded phenomenon of tendons caused a lower flexural capacity but higher ductility. The proposed modeling method for segmental joints and the developed muti-node slipping cable element for unbonded tendons can efficiently capture their mechanical behaviors. The effectiveness of the proposed model for the predictions of tendon stress increments, flexural capacity, and failure modes was fully verified against the experimental tests.
2. The flexural performances of the full-scale 37.5 m span PSCBs prestressed with three different prestressing schemes were compared. The PSCB with internal bonded steel tendons had the highest flexural capacity and ductility. The results for the PSCB with externally prestressed steel tendons were lowest. Compared with the externally prestressing scheme, with the same prestressing reinforcement ratio, the PSCB with internal unbonded CFRP tendons improved the flexural capacity by 54.6% and ductility indices by 8.9%. The internal unbonded CFRP tendon was a competitive alternative solution to the external steel tendon for the PSCB.
3. The parametric study showed that the loading types, span-to-depth ratios, and prestressing reinforcement ratios all had certain influences on the flexural behaviors of the PSCB with internal unbonded CFRP tendons. Under four-point loading states, more joints opened at the ultimate states, which was different from the analysis results of scaled specimens. The tendon stress increments and the flexural capacity were greater than those of the other loading conditions. The larger span-to-depth ratio *L*/*d*_p_ was not conducive to the flexural capacity. With *L*/*d*_p_ increasing by 23%, the flexural capacity decreased by about 38.6% and the tendon stress increment decreased by about 15.7%. With *ρ*_p_ increasing by 65.4%, the flexural capacity increased by 88.7%. However, the ultimate stress increment and the structural ductility decreased by 25.2% and 29.6%, respectively.

## Figures and Tables

**Table 1:** Properties of material used in specimens C2 and C4.

Type	Diameter (mm)	Area (mm^2^)	*f*_y_ (MPa)	*f*_u_ (MPa)	E (GPa)
Steel rebar	12.0	113.0	534	587	200
Steel rebar	10.0	78.5	489	538	200
CFRP tendon	12.9	126.7	—	2450	145

**Table 2:** Material properties of Beam-1 and Beam-2.

Type	Area (mm^2^)	*f*_y_ (MPa)	*f*_u_ (MPa)	E (GPa)	Post-Tensioning Stress (MPa)
Steel rebar ϕ12	113.0	350	380	200	-
Steel tendon	2100	1748	1860	195	1000
CFRP tendon	2100	-	2450	145	1000

**Table 3:** Comparisons of indices for PSCB.

Beam ID	∆*σ_p_* (MPa)	*σ_pu_* (MPa)	M*_y_* (10^3^ kN·m)	M*_u_* (10^3^ kN·m)	∆*_y_* (mm)	∆*_u_* (mm)	*μ* = ∆*_u_*/∆*_y_*
Beam-1	505	1486	10.2	22.9	18	524	29.1
Beam-2	552	1536	14.1	35.4	20	633	31.7
Beam-3	879	1860	45.5	47.2	20	724	36.2

## Data Availability

Not applicable.
